# UAlpha40: A comprehensive dataset of Urdu alphabet for Pakistan sign language

**DOI:** 10.1016/j.dib.2025.111342

**Published:** 2025-01-28

**Authors:** Sameena Javaid, Shahood Sajid, Yusra Khan Baloch

**Affiliations:** Department of Computer Science, School of Engineering Sciences, Bahria University, Karachi, Pakistan

**Keywords:** Sign language, Urdu alphabets, Finger spelling, Pakistan sign language, Computer Vision

## Abstract

Language bridges the gap of communication, and Sign Language (SL) is a native language among vocal and mute community. Every region has its own sign language. In Pakistan, Urdu Sign Language (USL) is a visual gesture language used by the deaf community for communication. The Urdu alphabet in Pakistan Sign Language consists not only of static gestures but also includes dynamic gestures. There are a total of 40 alphabets in Urdu sign language, with 36 being static and 4 being dynamic. While researchers have focused on the 36 static gestures, the 4 dynamic gestures have been overlooked. Additionally, there remains a lack of advancements in the development of Pakistan Sign Language (PSL) with respect to Urdu alphabets. A dataset named UAlpa40 has been compiled, comprising 22,280 images, among which 2,897 are originally created and 19,383 are created through noise or augmentation, representing the 36 static gestures and 393 videos representing the 4 dynamic gestures, completing the set of 40 Urdu alphabets. The standard gestures for USL are published by the Family Educational Services Foundation (FESF) for the deaf and mute community of Pakistan. This dataset was prepared in real-world environments under expert supervision, with volunteers ranging from males to females aged 20 to 45. This newly developed dataset can be utilized to train vision-based deep learning models, which in turn can aid in the development of sign language translators and finger-spelling systems for USL.

Specifications Table

**Table:** 

Subject	Computer Vision, Image Processing, Machine Learning, Pattern Recognitions, Human Computer Interaction, Video Processing
Specific subject area	Vision Based Pakistan Sign Language Urdu Alphabets Recognition
Type of data	Image files of JPG and video files of MP4 format.
Data collection	Three cameras were used to capture images and record videos. Camera 1 was EOS M50 Canon, Camera 2 was EOS 700D Canon and Camera 3 was D5100 Nikon. Three 500 W Adjustable Ultra-Bright LED Floodlights were used.
Data source location	Computer Science Department, School of Engineering and Applied Sciences, Bahria University
Data accessibility	Repository name: Mendeley Data Data identification number: 10.17632/3pvnnckxyb.1 Direct URL to data: https://data.mendeley.com/datasets/3pvnnckxyb/3
Related research article	Nil

## 1 Value of the Data

• This dataset is unique as it comprises of 22,280 36 static gestures and 393 videos for 4 dynamic gestures.
• The proposed dataset is complete; hence, it can be used to generate sentences.
• The proposed dataset can be utilized to develop a highly effective application, which then can be used to bridge the gap between hearing-impaired and normal hearing people.
• The proposed dataset can assist relevant researchers to create a product to cater the needs of deaf people.

## 2 Background

Sign Language is a native language of deaf people, it is just like a natural language which meets all social norms and mental functions like spoken language. However, the medium of communication differs from vocal auditory spoken languages. Sign language are visio-temporal constructs which conveys meanings through body movements and gestures, specifically hand gestures, head movements, upper body motions and facial expression [1].

As formerly, every country has its explicit Sign Language like: Chinese Sign Language, British Sign Language. In Pakistan, Urdu is the native language, also the language of deaf and mute people. Corpus of Pakistan Sign Language (PSL) may use some English alphabets also, but mainly the whole vocabulary is ruled and dominated by Urdu as a visual gesture language. However, publicly available dataset of Pakistan Sign Language is rare. Owing the lack of publicly available datasets, scientific research becomes hard. Similarly, with the advancements of real time applications, continuous sign language has immense importance due to its complex nature and video processing needs [2]. In this current dataset, we have recorded dynamic gestures for Urdu characters.

## 3 Data Description

In this paper, we introduced a publicly available Pakistan Sign Language dataset comprised of 22,280 images, among which 2897 are originally created and 19,383 are created through noise or augmentation, representing the 36 static gestures and 393 videos for 4 dynamic gestures [3]. Table 1 shows the Pakistan Sign Language alphabets in Roman English, Urdu with their number of samples. Primary objective of this dataset is to serve as new benchmark in Pakistan Sign Language with unique feature of dynamic gestures. It aims to facilitate researchers in the field of HCI, computer vision, machine learning and deep learning communities. This dataset is not only based on static hand gestures but also dynamic gestures.

**Table 1:** Pakistan Sign Language Alphabets with their names in Urdu and number of samples.

Alphabet Name in English	Alphabet Name in Urdu	Number of Samples	Number of Augmented Samples	Alphabet Name in English	Alphabet Name in Urdu	Number of Samples	Number of Augmented Samples
Alif mad	آ	105	NA	Suad	ص	82	602
Alif	ا	81	579	Zuad	ض	80	506
Bay	ب	82	608	Tuey	ط	80	506
Pay	پ	81	584	Zuey	ظ	80	506
Tay	ت	81	585	Ain	ع	80	506
Taay	ٹ	81	578	Ghain	غ	80	506
Say	ث	81	555	Fay	ف	80	506
Jeem	ج	99	NA	Kaf	ق	80	506
Chay	چ	81	547	Kiaf	ک	80	506
1-Hay	ح	81	578	Gaaf	گ	80	506
Khay	خ	80	554	Lam	ل	80	506
Dal	د	78	506	Meem	م	80	506
Daal	ڈ	81	578	Nuun	ن	80	501
Zaal	ذ	79	530	Nuungh	ں	80	506
Ray	ر	82	602	Wao	و	80	489
Aray	ڑ	102	NA	Hay	ہ	87	NA
Zay	ز	82	602	Dochahay	ھ	80	481
Zaey	ژ	82	602	Hamza	ء	79	482
Seen	س	82	602	Cyeh	ى	80	482
Sheen	ش	81	602	Byeh	ے	8	482

**Fig. 1:**
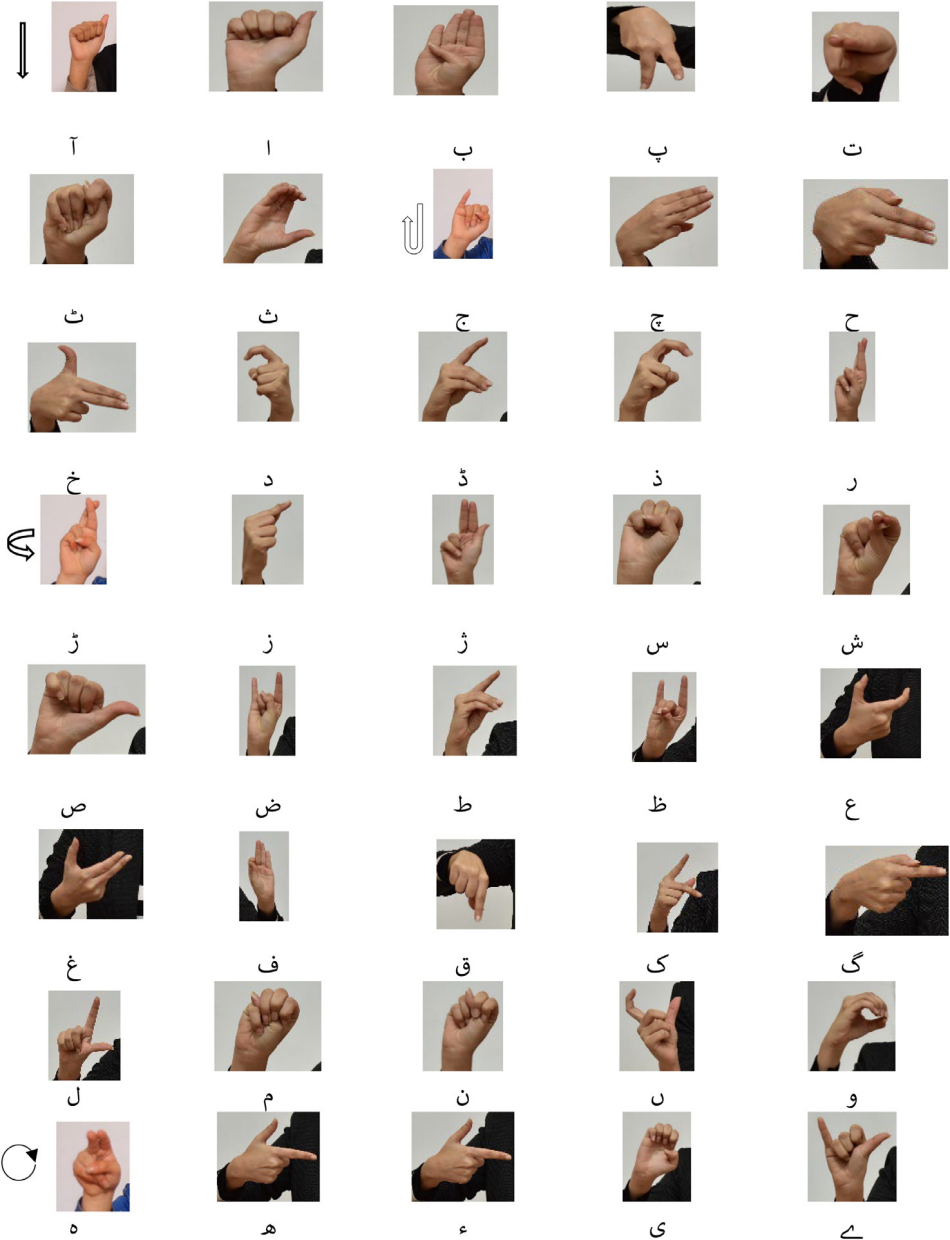
Urdu Character Set for Pakistan Sign Language.

Fig. 1 elaborates all signs for Pakistan Sign Language Urdu character set. To our knowledge, there is no publicly available dataset containing dynamic alphabets. The current dataset is notable for its broad vocabulary, high-quality recordings, and unique approach to recognizing dynamic hand movements.

## 4 Experimental Design, Materials and Methods

This section elaborates the experimental design, materials and methods used to collect the dataset. Section 4.1 explains the equipment used, its setup and configurations used. Then in Section 4.2 subjects and procedures are discussed. And finally in Section 4.3 already existing dataset are deliberated and compared.

### 4.1 Equipment and setup

The complete dataset was recorded with three cameras. The first camera was a versatile Canon EOS M50, the second a Canon EOS 700D, and the third a Nikon D5–100. We used three 500 W Adjustable Ultra-Bright LED Floodlights for illumination, with one spotlighting the subject and the other two providing background lighting and reducing shadows. Throughout the dataset production process, we used consistent cold lighting with a stable color temperature and either a white projection screen or a white background wall as the backdrop.

Images dataset was collected using Canon 700D and Nikon D5–700 with the configuration of lenses 50 mm and 40 mm respectively with auto AI and manual focus in the JPG format with the resolution of 4928 × 3264 and 5184 × 2912 likewise. Table 2 elaborates the details of equipment and image quality used for 36 classes of Static hand gestures from Urdu Sign Language.

**Table 2:** Details of equipment and Images for static signs.

FEATURES	CAMERA 1	CAMERA 2
Name of camera	CANON EOS-700D	NIKON D5–100
Dimension of image	4928×3264	5184×2912
Dots per Inches	300 dpi	72 dpi
Exposure time	1/100 s	1/30 s
ISO speed	ISO-400	ISO-2200
Focal length	50mm	40mm
Flash	No flash	No flash

Similarly, high-definition videos were captured using the Canon M50 and Canon 700D cameras, both with a frame size of 1920 × 1087 pixels and recorded at frames per second. The videos were saved in MP4 format. Table 3 provides detailed information about the videos recorded for the 04 dynamic hand gestures used in Urdu Sign Language.

**Table 3:** Details of equipment and videos for dynamic signs.

FEATURES	CAMERA 1	CAMERA 2
Name of camera	CANON EOS-M50	CANON EOS-700D
Frame Width	1920	1920
Frame Height	1087	1087
Data Rate	67589kbps	58473kbps
Total Bit Rate	67589kbps	58473kbps
Frame Rate	59.94 frames per second	59.94 frames per second

### 4.2 Subjects and procedures

With the approval of the BUKC Ethical Review Committee (ERC), we recorded 110 individuals performing gestures representing 40 Urdu alphabets in Pakistan Sign Language. The dataset was collected over approximately 20 days in June 2024. Each participant contributed 36 static images and 4 dynamic videos. The participants, both male and female, ranged in age from 20 to 45 years old and came from a variety of linguistic and educational backgrounds, including university students, professionals, and people with varying degrees of sign language proficiency. This diversity ensures a broad dataset that captures natural variances in gesture articulation.

During the photograph sessions, participants were instructed to maintain a neutral facial expression and face the camera directly. After each set of images, we reviewed them to ensure there were no movements, blinks, or changes in expression, and to confirm that all necessary images were captured with the desired quality. Although subjects' clothing was not standardized, and slight variations in posture and facial expressions occurred, all other aspects of the data collection process were carefully controlled.

When recording dynamic gestures, the speed of each individual's actions varied, resulting in video lengths ranging from 1 to 2 ss. Given that the hands were treated as the Region of Interest (ROI), backgrounds varied significantly due to differences in clothing colors and patterns, and individual differences in skin tone and facial features were also noted. These variations were intentionally included to support a more rigorous evaluation of algorithms, contributing to the dataset's robustness.

To further enhance the dataset's diversity, we applied several augmentation techniques, including JPG compression, Gaussian blur, speckle noise, Poisson noise, brightness adjustments, contrast changes, erosion, sharpening, flipping, salt-and-pepper noise, and Gaussian noise.

### 4.3 Comparison to datasets

Several researchers have worked on automating Pakistan Sign Language (PSL) computationally, but none of their work has been made available online for open access. In the early 2000s, the Deaf Reach Program, run by the Pakistan Sign Language organization, created a significant repository of 5000 static and dynamic signs [4]. This was a major advancement for the deaf and mute community. However, this repository was compiled with a single signer in a controlled environment, which limits its applicability for vision-based deep learning models that require extensive and complex real-world data [5].

Recently, from 2019 to 2024, a few researchers have started to publish datasets aimed at computer vision, machine learning, and deep learning in this domain. In 2019, Saad Butt and his colleagues released the Pakistan Sign Language Dataset (OpenPose) on Kaggle for automatic sign recognition. This dataset includes over 2000 images of 37 Urdu alphabets and 700 images of commonly used Urdu words, all captured with a webcam. The data were collected from 9 different individuals. The dataset is available as a JSON file, which contains skeletal key points for each image, utilizing the OpenPose library for pose detection. We found this dataset to be less challenging in terms of data robustness and its relevance to the domain [6].

Similarly, in 2020 Hand gesture recognition-Dataset is publicly available through Google drive by Abdullah Mujahid and fellows, this dataset consisted of 216 images for 5 classes, every class holds approximate 42 images and represents a finger pointing in terms of one, two, three, four and five. Dataset holds the labelled data into text file format, consisting of class ID and class category to which it belongs. This dataset was also compiled using a single individual which also makes it less robust, and the overall scope of the data is very limited [7]. Later in 2021, Ali Imran and his team published a dataset on Mendeley, which featured 40 images of a single hand configuration captured from multiple angles using a webcam. This dataset focused on static Urdu alphabets, covering 37 different classes with images from a single subject [8].

Many existing datasets of recent years of 2022 to 2024, focus solely on static sign language gestures, typically captured as still images [[9], [10], [11]]. Unfortunately, none of these datasets include dynamic gestures in video format. The limited scope and variety of these datasets can affect system efficiency, though they do offer some flexibility. While there are a few datasets that do feature dynamic gestures, they often vary in their approach and scope, leading to differences in how these dynamic gestures are represented and analysed [[12], [13], [14], [15]].

In our current dataset, we have tried to make our dataset more robust and challenging. By recording 100 individuals for 40 classes of static as well as dynamic gestures. Every individual had different attire, flair, speed, body language, formation of signs and unique hand and general features. Our dataset is not only domain specific for signers only, but researchers also working with video frame segmentation, video frame selection, augmentation techniques, preprocessing and many other visions based deep learning research can get equal benefits, which will further lead to proficient automatic sign language recognition. The current dataset has been visually reviewed and collected with the assistance of domain experts. The data has been cleaned to remove any erroneous signs, ensuring that only accurate and relevant information remains. A balanced distribution among the classes has been maintained, with a proper labelling scheme applied to ensure consistency and reliability.

## Limitations

Consequently, there is still a room for improvement and dataset can be extended in terms of more gestures, varied and complex backgrounds, noise variation, orientation and scaling variations, more number of participants etc.

## Ethics Statement

This data collection involved human subjects, and written consent was obtained from all participants. The privacy of all individuals has been carefully safeguarded.

## Credit Author Statement

**Sameena Javed:** Conceptualization, Data curation, Supervision, Writing Original draft. **Shahood Sajid:** Writing Original draft, Writing – Review & Editing. **Yusra Khan Baloch:** Writing – Review & Editing.

## Data Availability

Mendeley DataUAlpha40: A Comprehensive Dataset of Urdu Alphabets for Pakistan Sign Language (Original data).

## Appendix Supplementary materials

Image, application 1
