# Supplementary material for: UAlpha40: A comprehensive dataset of Urdu alphabet for Pakistan sign language
Source: Data Brief. 2025 Jan 28;59:111342. doi: 10.1016/j.dib.2025.111342 (PMC11848795; doi:10.1016/j.dib.2025.111342)
Supplement: Supplementary file 1 [file mmc1.pdf]

Bahria University
Discovering Knowledge

PhD-12

Ethical Review Form
Part-I

This Form is designed to ensure that the departmental research operates an ethical review process that falls within the University guidelines. Any scholar undertaking research on live human subjects needs to fill this Form. If all questions in this Form are answered 'No', ethical approval shall automatically be granted. In such case Part-II of this Form shall not be filled by the scholar. If any of the questions is answered 'Yes', the scholar shall be required to fill Part-II of this Form. This part of the Form shall be reviewed by the Ethical Review Committee (ERC). Scholars shall be informed of the decision of the ERC as soon as possible. If needed, the scholar may be asked to submit further information and appear before the ERC for discussion meeting.

Scholar's Name:	Sameena Javaid
Reg. No:	36798 (02-284172-002)
Faculty/Department	Computer Science Department (Karachi)
Programme:	PhD (Computer Science)
Title of the Thesis:	Dynamic Sign Language Recognition Using Deep Learning
Principal Supervisor:	Dr. S. Safdar Ali Rizvi

Name of Supervisor:	Dr. Syed Safdar Ali Rizvi
Faculty/Department	Computer Science Department
Designation:	Associate Professor

Name of Co-Supervisor:	—x—
Faculty/Department	—x—
Designation:	—x—

Aim/purpose of study, source(s), Method(s) of Data Collection, benefits of study, duration of the study (*not more than one page, use extra sheet if required*).
* See Annex-A for details.

Scanned with CamScanner

Bahria University
Discovering Knowledge

Please answer all of the questions below by ticking (✓) 'Yes' or 'No' in the box provided

		Yes	No
1.	Does the study involve participants who are particularly vulnerable or unable to give informed consent? (e.g. people under the age of 18, people with disabilities etc.)		✓
2.	Will it be necessary for the participants to take part in the study without their knowledge and consent?		✓
3.	Does the study involve audio or visual recording of people in public places?		✓
4.	Will the study involve the discussion of sensitive topics? (e.g. sexual activity, drug use, illegal activities, death, whistle-blowing etc.)		✓
5.	Does the research involve the use of drugs, radiation agents experimental surgical / harmful procedures, blood or tissues samples		✓
6.	Is physical pain or psychological stress being part of this research work is likely to cause harm or negative consequences to the participants?		✓
7.	Will the study involve prolonged or repetitive testing on the participants?		✓
8.	Will financial inducements be involved in the study and (other than expenses) be offered to participants?		✓
9.	Will the study involve recruitment of patients or staff?		✓

If you have answered 'yes' to any of these questions, please fill in Part-II as well. Otherwise, simply submit Part-I of the form.

**Scholar**	*Verified by* **Principal Supervisor**
Signature: [signature]	Signature: [signature]
Date: 6th January 2020	Date: 6/01/2020

Scanned with CamScanner

Annexure A

PURPOSE OF STUDY

The title of the research study is "Dynamic Sign Language Recognition using Deep Learning", which implies that the area of research is Sign Language (SL), which is the language of deaf and mute people. Sign Language is unique for every region. Further Sign Language recognition is not trivial as each person may pose the same gesture differently. At the same time to identify a single gesture, Manual and Non Manual parameters are both important. Manual gestures are based on Shape, location movement and orientation of hand and Non-Manual gestures are position and movement of head and torso and most important Facial Expression (FE).

Pakistan Sign Language (PSL) is a subject of studies of many researchers but limited literature was found along with deficiency in publically available dataset(s). Likewise manual and non-manual parameters in dynamic sign language recognition with a unique URDU grammatical structure needs attention for our community and assistance for special persons with speech impairment disability of Pakistani individuals to make them more valuable part of our society.

SOURCES

- Data set is collected by around 300+ individuals, among which several are mute (special persons with speech and hearing disability)
- All individuals have given data volunteer with complete consent and willingness
- All individuals are adults and having age between 18 to 50 years
- All disabled persons are well informed about the dataset they are giving, through sign language interpreters
- Data Set consist of following signs:
    - A to Z english alphabets
    - Alif to Yeh urdu alphabets
    - 31 signs related to numbers and counting
    - 38 adjectives
- Organizations in contact are:
    - Special Children School (Bahria Karsaz).
    - Deaf Reach
    - FESF (psl.org.pk)
    - BridgingSigns (Project of AWAD)
    - ABSA

METHODS OF DATA COLLECTION

Data set is recorded in form of:

- Pictures (for static gestures)
- Videos (for dynamic gestures)

Cameras which were used are:

- Canon m 50
- Canon 700D

Scanned with CamScanner

Bahria University
Discovering Knowledge

B. Scholar is also required to do the following:

1. Prepare an information letter for their participants. The letter needs to introduce the scholar and provide a simple explanation of the research. It needs to make clear what the participants are asked to do, how long the research work will take. The letter shall also include whether it will be/not be anonymous and/or confidential and who will have access to the data.

2. Prepare a Consent Form containing the phrases "I agree to participate in the research work PKSL Dataset and I understand my information will be/will not be treated as confidential, and/or anonymous."

Scholar

Name: Sameera Javaid

Signature: [signature]

Date: 06-01-20

Scanned with CamScanner

Empty Consent

PARTICIPANT CONSENT FORM

Scholar statement

We are inviting you to participate in an activity of data set collection regarding Pakistan Sign Language (PSL) recognition. This activity is related to PhD thesis work naming "Dynamic Sign Language Recognition using Deep Learning". The complete activity will not take more than one hour. It consists of static and dynamic gestures recording for PSL using hands, head, torso and facial expression of individuals.

By participating you would be helping us to work for deaf and mute people in Pakistan and Worldwide. This dataset will be used only for research and publication purposes.

Participant Statement

I agree to participate in this study under the conditions set above.

Participant Name: ______________________________

Participant Signatue: ___________________________

Project Contacts

PhD Scholar

Sameena Javaid

javaidsameena@gmail.com

Thesis Supervisor

Dr. Syed Safdar Ali Rizvi

Associate Professor

Department of Computer Science

Bahria University Karachi Campus

Scanned with CamScanner

TO WHOM IT MAY CONCERN

I am writing to inform you that I have completed my evaluation of Sign Language dataset submitted and prepared by Ms. Sameena Javaid, student of PhD (Computer Science Department Bahria University Karachi Campus). And I am pleased to inform that it is valid and of high quality.

I have carefully reviewed the data she has collected and verified that it met all of the necessary criteria for reliability, validity, and relevance. I understand that collecting data can be a time consuming and challenging process, and I commend you on your effort, and appreciate your contribution to the advancement of knowledge and the improvement of research practices.

Do not hesitate to contact us, if you have any question or concern.

Nazia Batool

Hearing Impaired Teacher

Bahria College Karsaz (Special Children School)

naziajawed83@gmail.com

Scanned with CamScanner

TO WHOM IT MAY CONCERN

I am writing to inform you that I have completed my evaluation of Sign Language dataset submitted and prepared by Ms. Sameena Javaid, student of PhD (Computer Science Department Bahria University Karachi Campus). And I am pleased to inform that it is valid and of high quality.

I have carefully reviewed the data she has collected and verified that it met all of the necessary criteria for reliability, validity, and relevance. I understand that collecting data can be a time consuming and challenging process, and I commend you on your effort, and appreciate your contribution to the advancement of knowledge and the improvement of research practices.

Do not hesitate to contact us, if you have any question or concern.

Zeeshan

Muahmmad Zeeshan

Sign Language Interpreter

NOWPDP - A Disability Inclusion Initiative

(A leading organization for disabled persons)

mzeeshan211393@gmail.com

Scanned with CamScanner

TO WHOM IT MAY CONCERN

I am writing to inform you that I have completed my evaluation of Sign Language dataset submitted and prepared by Ms. Sameena Javaid, student of PhD (Computer Science Department Bahria University Karachi Campus). And I am pleased to inform that it is valid and of high quality.

I have carefully reviewed the data she has collected and verified that it met all of the necessary criteria for reliability, validity, and relevance. I understand that collecting data can be a time consuming and challenging process, and I commend you on your effort, and appreciate your contribution to the advancement of knowledge and the improvement of research practices.

Do not hesitate to contact us, if you have any question or concern.

AHayat

Asad Hayat

Hearing Impaired Teacher

Anjuman Behbood-e-Samat-e-Atfal (ABSA)

School and Dollege for the Deaf & NGO

asadhayat203@gmail.com

Scanned with CamScanner

PARTICIPANT CONSENT FORM

Scholar Statement

We are inviting you to participate in an activity of data set collection regarding Pakistan Sign Language (PSL) recognition. This activity is related to PhD thesis work naming "Dynamic Sign Language Recognition using Deep Learning". The complete activity will not take more than 1 hour. It consists of Static and Dynamic gestures recording of PSL using hands, head, torso and facial expression of individuals.

By participating you would be helping us to work for deaf and mute people in Pakistan and Worldwide. This dataset will be used only for research and publication purposes. Your identity will remain confidential.

Participant Statement

I agree to participate in this study under the conditions set above.

Participant Name: Ali Abbas Junejo

Participant Signature: ____________________

Project Contacts

PhD Scholar

Sameena Javaid

javaidsameena@gmail.com

Thesis Supervisor

Dr. Syed Safdar Ali Rizvi

Associate Professor

Department of Computer Science

Bahria University Karachi Campus

Scanned with CamScanner

PARTICIPANT CONSENT FORM

Scholar Statement

We are inviting you to participate in an activity of data set collection regarding Pakistan Sign Language (PSL) recognition. This activity is related to PhD thesis work naming "Dynamic Sign Language Recognition using Deep Learning". The complete activity will not take more than 1 hour. It consists of Static and Dynamic gestures recording of PSL using hands, head, torso and facial expression of individuals.

By participating you would be helping us to work for deaf and mute people in Pakistan and Worldwide. This dataset will be used only for research and publication purposes. Your identity will remain confidential.

Participant Statement

I agree to participate in this study under the conditions set above.

Participant Name: Noreen Bano

Participant Signature: ____________________

Project Contacts

PhD Scholar

Sameena Javaid

javaidsameena@gmail.com

Thesis Supervisor

Dr. Syed Safdar Ali Rizvi

Associate Professor

Department of Computer Science

Bahria University Karachi Campus

Scanned with CamScanner

PARTICIPANT CONSENT FORM

Scholar Statement

We are inviting you to participate in an activity of data set collection regarding Pakistan Sign Language (PSL) recognition. This activity is related to PhD thesis work naming "Dynamic Sign Language Recognition using Deep Learning". The complete activity will not take more than 1 hour. It consists of Static and Dynamic gestures recording of PSL using hands, head, torso and facial expression of individuals.

By participating you would be helping us to work for deaf and mute people in Pakistan and Worldwide. This dataset will be used only for research and publication purposes. Your identity will remain confidential.

Participant Statement

I agree to participate in this study under the conditions set above.

Participant Name: Laique Afzal

Participant Signature: Laiq.

Project Contacts

PhD Scholar

Sameena Javaid

javaidsameena@gmail.com

Thesis Supervisor

Dr. Syed Safdar Ali Rizvi

Associate Professor

Department of Computer Science

Bahria University Karachi Campus

Scanned with CamScanner

PARTICIPANT CONSENT FORM

Scholar Statement

We are inviting you to participate in an activity of data set collection regarding Pakistan Sign Language (PSL) recognition. This activity is related to PhD thesis work naming "Dynamic Sign Language Recognition using Deep Learning". The complete activity will not take more than 1 hour. It consists of Static and Dynamic gestures recording of PSL using hands, head, torso and facial expression of individuals.

By participating you would be helping us to work for deaf and mute people in Pakistan and Worldwide. This dataset will be used only for research and publication purposes. Your identity will remain confidential.

Participant Statement

I agree to participate in this study under the conditions set above.

Participant Name: Aarish Raza

Participant Signature: Aarish Raza

Project Contacts

PhD Scholar

Sameena Javaid

javaidsameena@gmail.com

Thesis Supervisor

Dr. Syed Safdar Ali Rizvi

Associate Professor

Department of Computer Science

Bahria University Karachi Campus

Scanned with CamScanner

PARTICIPANT CONSENT FORM

Scholar Statement

We are inviting you to participate in an activity of data set collection regarding Pakistan Sign Language (PSL) recognition. This activity is related to PhD thesis work naming "Dynamic Sign Language Recognition using Deep Learning". The complete activity will not take more than 1 hour. It consists of Static and Dynamic gestures recording of PSL using hands, head, torso and facial expression of individuals.

By participating you would be helping us to work for deaf and mute people in Pakistan and Worldwide. This dataset will be used only for research and publication purposes. Your identity will remain confidential.

Participant Statement

I agree to participate in this study under the conditions set above.

Participant Name: Naveed sheikh

Participant Signature: N.sheikh

Project Contacts

PhD Scholar

Sameena Javaid

javaidsameena@gmail.com

Thesis Supervisor

Dr. Syed Safdar Ali Rizvi

Associate Professor

Department of Computer Science

Bahria University Karachi Campus

Scanned with CamScanner

PARTICIPANT CONSENT FORM

Scholar Statement

We are inviting you to participate in an activity of data set collection regarding Pakistan Sign Language (PSL) recognition. This activity is related to PhD thesis work naming "Dynamic Sign Language Recognition using Deep Learning". The complete activity will not take more than 1 hour. It consists of Static and Dynamic gestures recording of PSL using hands, head, torso and facial expression of individuals.

By participating you would be helping us to work for deaf and mute people in Pakistan and Worldwide. This dataset will be used only for research and publication purposes. Your identity will remain confidential.

Participant Statement

I agree to participate in this study under the conditions set above.

Participant Name: Taha Ahmed Khan

Participant Signature: [signature]

Project Contacts

PhD Scholar

Sameena Javaid

javaidsameena@gmail.com

Thesis Supervisor

Dr. Syed Safdar Ali Rizvi

Associate Professor

Department of Computer Science

Bahria University Karachi Campus

Scanned with CamScanner

PARTICIPANT CONSENT FORM

Scholar Statement

We are inviting you to participate in an activity of data set collection regarding Pakistan Sign Language (PSL) recognition. This activity is related to PhD thesis work naming "Dynamic Sign Language Recognition using Deep Learning". The complete activity will not take more than 1 hour. It consists of Static and Dynamic gestures recording of PSL using hands, head, torso and facial expression of individuals.

By participating you would be helping us to work for deaf and mute people in Pakistan and Worldwide. This dataset will be used only for research and publication purposes. Your identity will remain confidential.

Participant Statement

I agree to participate in this study under the conditions set above.

Participant Name: Qalb-e-Raza.

Participant Signature: Qalb Raza

Project Contacts

PhD Scholar

Sameena Javaid

javaidsameena@gmail.com

Thesis Supervisor

Dr. Syed Safdar Ali Rizvi

Associate Professor

Department of Computer Science

Bahria University Karachi Campus

Scanned with CamScanner

PARTICIPANT CONSENT FORM

Scholar Statement

We are inviting you to participate in an activity of data set collection regarding Pakistan Sign Language (PSL) recognition. This activity is related to PhD thesis work naming "Dynamic Sign Language Recognition using Deep Learning". The complete activity will not take more than 1 hour. It consists of Static and Dynamic gestures recording of PSL using hands, head, torso and facial expression of individuals.

By participating you would be helping us to work for deaf and mute people in Pakistan and Worldwide. This dataset will be used only for research and publication purposes. Your identity will remain confidential.

Participant Statement

I agree to participate in this study under the conditions set above.

Participant Name: Sufiyan

Participant Signature: ______________________

Project Contacts

PhD Scholar

Sameena Javaid

javaidsameena@gmail.com

Thesis Supervisor

Dr. Syed Safdar Ali Rizvi

Associate Professor

Department of Computer Science

Bahria University Karachi Campus

Scanned with CamScanner

PARTICIPANT CONSENT FORM

Scholar Statement

We are inviting you to participate in an activity of data set collection regarding Pakistan Sign Language (PSL) recognition. This activity is related to PhD thesis work naming "Dynamic Sign Language Recognition using Deep Learning". The complete activity will not take more than 1 hour. It consists of Static and Dynamic gestures recording of PSL using hands, head, torso and facial expression of individuals.

By participating you would be helping us to work for deaf and mute people in Pakistan and Worldwide. This dataset will be used only for research and publication purposes. Your identity will remain confidential.

Participant Statement

I agree to participate in this study under the conditions set above.

Participant Name: Hadi

Participant Signature: Hadi

Project Contacts

PhD Scholar

Sameena Javaid

javaidsameena@gmail.com

Thesis Supervisor

Dr. Syed Safdar Ali Rizvi

Associate Professor

Department of Computer Science

Bahria University Karachi Campus

Scanned with CamScanner

PARTICIPANT CONSENT FORM

Scholar Statement

We are inviting you to participate in an activity of data set collection regarding Pakistan Sign Language (PSL) recognition. This activity is related to PhD thesis work naming "Dynamic Sign Language Recognition using Deep Learning". The complete activity will not take more than 1 hour. It consists of Static and Dynamic gestures recording of PSL using hands, head, torso and facial expression of individuals.

By participating you would be helping us to work for deaf and mute people in Pakistan and Worldwide. This dataset will be used only for research and publication purposes. Your identity will remain confidential.

Participant Statement

I agree to participate in this study under the conditions set above.

Participant Name: Rao Hashir

Participant Signature: R.Hashir

Project Contacts

PhD Scholar

Sameena Javaid

javaidsameena@gmail.com

Thesis Supervisor

Dr. Syed Safdar Ali Rizvi

Associate Professor

Department of Computer Science

Bahria University Karachi Campus

Scanned with CamScanner

PARTICIPANT CONSENT FORM

Scholar Statement

We are inviting you to participate in an activity of data set collection regarding Pakistan Sign Language (PSL) recognition. This activity is related to PhD thesis work naming "Dynamic Sign Language Recognition using Deep Learning". The complete activity will not take more than 1 hour. It consists of Static and Dynamic gestures recording of PSL using hands, head, torso and facial expression of individuals.

By participating you would be helping us to work for deaf and mute people in Pakistan and Worldwide. This dataset will be used only for research and publication purposes. Your identity will remain confidential.

Participant Statement

I agree to participate in this study under the conditions set above.

Participant Name: Abdullah

Participant Signature: Abdullah

Project Contacts

PhD Scholar

Sameena Javaid

javaidsameena@gmail.com

Thesis Supervisor

Dr. Syed Safdar Ali Rizvi

Associate Professor

Department of Computer Science

Bahria University Karachi Campus

Scanned with CamScanner

PARTICIPANT CONSENT FORM

Scholar Statement

We are inviting you to participate in an activity of data set collection regarding Pakistan Sign Language (PSL) recognition. This activity is related to PhD thesis work naming "Dynamic Sign Language Recognition using Deep Learning". The complete activity will not take more than 1 hour. It consists of Static and Dynamic gestures recording of PSL using hands, head, torso and facial expression of individuals.

By participating you would be helping us to work for deaf and mute people in Pakistan and Worldwide. This dataset will be used only for research and publication purposes. Your identity will remain confidential.

Participant Statement

I agree to participate in this study under the conditions set above.

Participant Name: Ayan

Participant Signature: Ayan 31/3/21

Project Contacts

PhD Scholar

Sameena Javaid

javaidsameena@gmail.com

Thesis Supervisor

Dr. Syed Safdar Ali Rizvi

Associate Professor

Department of Computer Science

Bahria University Karachi Campus

Scanned with CamScanner

PARTICIPANT CONSENT FORM

Scholar Statement

We are inviting you to participate in an activity of data set collection regarding Pakistan Sign Language (PSL) recognition. This activity is related to PhD thesis work naming "Dynamic Sign Language Recognition using Deep Learning". The complete activity will not take more than 1 hour. It consists of Static and Dynamic gestures recording of PSL using hands, head, torso and facial expression of individuals.

By participating you would be helping us to work for deaf and mute people in Pakistan and Worldwide. This dataset will be used only for research and publication purposes. Your identity will remain confidential.

Participant Statement

I agree to participate in this study under the conditions set above.

Participant Name: Mubashir

Participant Signature: Mubashir

Project Contacts

PhD Scholar

Sameena Javaid

javaidsameena@gmail.com

Thesis Supervisor

Dr. Syed Safdar Ali Rizvi

Associate Professor

Department of Computer Science

Bahria University Karachi Campus

Scanned with CamScanner

PARTICIPANT CONSENT FORM

Scholar Statement

We are inviting you to participate in an activity of data set collection regarding Pakistan Sign Language (PSL) recognition. This activity is related to PhD thesis work naming "Dynamic Sign Language Recognition using Deep Learning". The complete activity will not take more than 1 hour. It consists of Static and Dynamic gestures recording of PSL using hands, head, torso and facial expression of individuals.

By participating you would be helping us to work for deaf and mute people in Pakistan and Worldwide. This dataset will be used only for research and publication purposes. Your identity will remain confidential.

Participant Statement

I agree to participate in this study under the conditions set above.

Participant Name: Shaheer Bin Yamin.

Participant Signature: S bin Yamin

Project Contacts

PhD Scholar

Sameena Javaid

javaidsameena@gmail.com

Thesis Supervisor

Dr. Syed Safdar Ali Rizvi

Associate Professor

Department of Computer Science

Bahria University Karachi Campus

Scanned with CamScanner

PARTICIPANT CONSENT FORM

Scholar Statement

We are inviting you to participate in an activity of data set collection regarding Pakistan Sign Language (PSL) recognition. This activity is related to PhD thesis work naming "Dynamic Sign Language Recognition using Deep Learning". The complete activity will not take more than 1 hour. It consists of Static and Dynamic gestures recording of PSL using hands, head, torso and facial expression of individuals.

By participating you would be helping us to work for deaf and mute people in Pakistan and Worldwide. This dataset will be used only for research and publication purposes. Your identity will remain confidential.

Participant Statement

I agree to participate in this study under the conditions set above.

Participant Name: Sharjeel Abbas

Participant Signature: Sharjeel

Project Contacts

PhD Scholar

Sameena Javaid

javaidsameena@gmail.com

Thesis Supervisor

Dr. Syed Safdar Ali Rizvi

Associate Professor

Department of Computer Science

Bahria University Karachi Campus

Scanned with CamScanner

PARTICIPANT CONSENT FORM

Scholar Statement

We are inviting you to participate in an activity of data set collection regarding Pakistan Sign Language (PSL) recognition. This activity is related to PhD thesis work naming "Dynamic Sign Language Recognition using Deep Learning". The complete activity will not take more than 1 hour. It consists of Static and Dynamic gestures recording of PSL using hands, head, torso and facial expression of individuals.

By participating you would be helping us to work for deaf and mute people in Pakistan and Worldwide. This dataset will be used only for research and publication purposes. Your identity will remain confidential.

Participant Statement

I agree to participate in this study under the conditions set above.

Participant Name: Meenal Hasija

Participant Signature: Meenal.

Project Contacts

PhD Scholar

Sameena Javaid

javaidsameena@gmail.com

Thesis Supervisor

Dr. Syed Safdar Ali Rizvi

Associate Professor

Department of Computer Science

Bahria University Karachi Campus

Scanned with CamScanner

PARTICIPANT CONSENT FORM

Scholar Statement

We are inviting you to participate in an activity of data set collection regarding Pakistan Sign Language (PSL) recognition. This activity is related to PhD thesis work naming "Dynamic Sign Language Recognition using Deep Learning". The complete activity will not take more than 1 hour. It consists of Static and Dynamic gestures recording of PSL using hands, head, torso and facial expression of individuals.

By participating you would be helping us to work for deaf and mute people in Pakistan and Worldwide. This dataset will be used only for research and publication purposes. Your identity will remain confidential.

Participant Statement

I agree to participate in this study under the conditions set above.

Participant Name: Maryam Shahid

Participant Signature: [signature]

Project Contacts

PhD Scholar

Sameena Javaid

javaidsameena@gmail.com

Thesis Supervisor

Dr. Syed Safdar Ali Rizvi

Associate Professor

Department of Computer Science

Bahria University Karachi Campus

Scanned with CamScanner

PARTICIPANT CONSENT FORM

Scholar Statement

We are inviting you to participate in an activity of data set collection regarding Pakistan Sign Language (PSL) recognition. This activity is related to PhD thesis work naming "Dynamic Sign Language Recognition using Deep Learning". The complete activity will not take more than 1 hour. It consists of Static and Dynamic gestures recording of PSL using hands, head, torso and facial expression of individuals.

By participating you would be helping us to work for deaf and mute people in Pakistan and Worldwide. This dataset will be used only for research and publication purposes. Your identity will remain confidential.

Participant Statement

I agree to participate in this study under the conditions set above.

Participant Name: Syed Owais Hassan

Participant Signature: ______________

Project Contacts

PhD Scholar

Sameena Javaid

javaidsameena@gmail.com

Thesis Supervisor

Dr. Syed Safdar Ali Rizvi

Associate Professor

Department of Computer Science

Bahria University Karachi Campus

Scanned with CamScanner

PARTICIPANT CONSENT FORM

Scholar Statement

We are inviting you to participate in an activity of data set collection regarding Pakistan Sign Language (PSL) recognition. This activity is related to PhD thesis work naming "Dynamic Sign Language Recognition using Deep Learning". The complete activity will not take more than 1 hour. It consists of Static and Dynamic gestures recording of PSL using hands, head, torso and facial expression of individuals.

By participating you would be helping us to work for deaf and mute people in Pakistan and Worldwide. This dataset will be used only for research and publication purposes. Your identity will remain confidential.

Participant Statement

I agree to participate in this study under the conditions set above.

Participant Name: Reeza Aftab

Participant Signature: Reeza.

Project Contacts

PhD Scholar

Sameena Javaid

javaidsameena@gmail.com

Thesis Supervisor

Dr. Syed Safdar Ali Rizvi

Associate Professor

Department of Computer Science

Bahria University Karachi Campus

Scanned with CamScanner

PARTICIPANT CONSENT FORM

Scholar Statement

We are inviting you to participate in an activity of data set collection regarding Pakistan Sign Language (PSL) recognition. This activity is related to PhD thesis work naming "Dynamic Sign Language Recognition using Deep Learning". The complete activity will not take more than 1 hour. It consists of Static and Dynamic gestures recording of PSL using hands, head, torso and facial expression of individuals.

By participating you would be helping us to work for deaf and mute people in Pakistan and Worldwide. This dataset will be used only for research and publication purposes. Your identity will remain confidential.

Participant Statement

I agree to participate in this study under the conditions set above.

Participant Name: Mahnoor Arif

Participant Signature: [signature]

Project Contacts

PhD Scholar

Sameena Javaid

javaidsameena@gmail.com

Thesis Supervisor

Dr. Syed Safdar Ali Rizvi

Associate Professor

Department of Computer Science

Bahria University Karachi Campus

Scanned with CamScanner
